# A fish protein hydrolysate alters fatty acid composition in liver and adipose tissue and increases plasma carnitine levels in a mouse model of chronic inflammation

**DOI:** 10.1186/1476-511X-12-143

**Published:** 2013-10-07

**Authors:** Bodil Bjørndal, Christ Berge, Marie Sannes Ramsvik, Asbjørn Svardal, Pavol Bohov, Jon Skorve, Rolf K Berge

**Affiliations:** 1 Institute of Clinical Science, University of Bergen, N-5020 Bergen, Norway; 2 Department of Heart Disease, Haukeland University Hospital, N-5021 Bergen, Norway

**Keywords:** Salmon protein, High-fat diet, Tumor necrosis factor α, Inflammation, Carnitine metabolism

## Abstract

**Background:**

There is growing evidence that fish protein hydrolysate (FPH) diets affect mitochondrial fatty acid metabolism in animals. The aim of the study was to determine if FPH could influence fatty acid metabolism and inflammation in transgene mice expressing human tumor necrosis factor alpha (hTNFα).

**Methods:**

hTNFα mice (C57BL/6 hTNFα) were given a high-fat (23%, w/w) diet containing 20% casein (control group) or 15% FPH and 5% casein (FPH group) for two weeks. After an overnight fast, blood, adipose tissue, and liver samples were collected. Gene expression and enzyme activity was analysed in liver, fatty acid composition was analyzed in liver and ovarian white adipose tissue, and inflammatory parameters, carnitine, and acylcarnitines were analyzed in plasma.

**Results:**

The n-3/n-6 fatty acid ratio was higher in mice fed the FPH diet than in mice fed the control diet in both adipose tissue and liver, and the FPH diet affected the gene expression of ∆6 and ∆9 desaturases. Mice fed this diet also demonstrated lower hepatic activity of fatty acid synthase. Concomitantly, a lower plasma INF-γ level was observed. Plasma carnitine and the carnitine precursor γ-butyrobetaine was higher in the FPH-group compared to control, as was plasma short-chained and medium-chained acylcarnitine esters. The higher level of plasma acetylcarnitine may reflect a stimulated mitochondrial and peroxisomal β-oxidation of fatty acids, as the hepatic activities of peroxisomal acyl-CoA oxidase 1 and mitochondrial carnitine palmitoyltransferase-II were higher in the FPH-fed mice.

**Conclusions:**

The FPH diet was shown to influence hepatic fatty acid metabolism and fatty acid composition. This indicates that effects on fatty acid metabolism are important for the bioactivity of protein hydrolysates of marine origin.

## Background

Dietary proteins and the bioactive peptides generated from proteins, either formed naturally in the gut or delivered in the diet as hydrolyzed proteins, may have a number of important effects beyond their role as sources of amino acids and energy [1]. Studies on fish peptides have demonstrated antihypertensive [2-5], antioxidant [6-9], and immunomodulating effects [10], as well as reparative properties in the intestine [11,12]. Hydrolyzed proteins from plant and fish have been demonstrated to alter the cholesterol and lipid metabolism in rodent studies, and to reduce plasma cholesterol and triglyceride levels [1,13-15]. In addition, we previously found a reduction in hepatic ∆5 and ∆6 desaturase mRNA expression in obese Zucker rats by a fish protein hydrolysate (FPH) diet [16].

There is an inter-organ transport of fatty acids, and major tissues in fatty acid metabolism from an energy point of view are the gut, white adipose tissue (WAT), liver and muscle. The liver plays a major role in the desaturation and elongation processes that determine the fatty acid composition during *de novo* lipogenesis. Studies have indicated that diet-induced alterations in the membrane phospholipid composition has important effects on inflammation, in particular through increased anti-inflammatory prostaglandin and resolvine production from n-3 polyunsaturated fatty acids (PUFAs) [17]. The ∆9 desaturase steroyl-CoA desaturase 1 (SCD1) is important for the generation of monounsaturated fatty acids during lipogenesis. It plays a role in inflammatory regulation, and its upregulation is correlated to many metabolic diseases including obesity and insulin resistance [18,19]. Previous studies indicate that dietary protein composition plays an important role in the regulation of fatty acid desaturation as well as cholesterol metabolism [14,20,21]. Thus, protein hydrolysate diets could potentially target lipogenesis and desaturation and thereby positively influence metabolic disturbances.

As hydrolyzed proteins have the ability to modulate both lipid metabolism, and inflammation processes, we investigated in the present study whether a 15% FPH diet high in glycine and taurine could counteract the effects of tumor necrosis factor alpha (TNFα) overexpression. Mice transgenic for human TNFα (hTNFα) have previously been shown to display dyslipidemia, altered lipid composition, and reduced activation of peroxisome proliferator-activated receptor alpha (PPARα) regulated genes [22]. In the present study, we show that the administration of FPH to hTNFα mice fed a high-fat diet affected fatty acid composition in liver and WAT with a concurrent increase in the fatty acid anti-inflammatory index. In addition, plasma carnitine and short-chained acylcarnitine esters, and hepatic peroxisomal fatty acid oxidation were increased, while lipogenesis was reduced.

## Results

### The effect of FPH on feed intake and blood lipids

TNFα-mice were fed high-fat diets with or without 15% FPH for two weeks. No changes were observed in fasting plasma or hepatic lipid levels (Table 1). There was a significantly higher weight gain, but no difference in feed efficiency in the FPH-group compared to control after two weeks feeding. Feed intake was higher in the FPH-group, and this could have caused the higher weight gain compared to control (Table 2).

**Table 1:** Fasting lipid levels in TNFα mice given a control- or a FPH-high-fat diet for two weeks

	**Plasma lipids**		**Hepatic lipids**	
**Treatment**	**Control**	**FPH**	**Control**	**FPH**
	**(mmol/L)**	**(mmol/L)**	**(μmol/g liver)**	**(μmol/g liver)**
Cholesterol	1.74 ± 0.18	1.71 ± 0.18	8.2 ± 1.3	7.7 ± 0.8
Triacylglycerol	0.50 ± 0.15	0.58 ± 0.10	25.3 ± 2.8	29.3 ± 4.6
Phospholipids	1.67 ± 0.23	1.66 ± 0.14	18.9 ± 0.78	19.0 ± 0.52

*Abbreviations*: *FPH* fish protein hydrolysate, *TNFα* tumor necrosis factor alpha.

Mean values ± SD are shown (n = 5).

Mann–Whitney analysis (p < 0.05) was used to determine if values were significantly different from control.

**Table 2:** Weight gain and feed intake in TNFα mice given control- or FPH-diets for two weeks

**Treatment**	**Weight gain**^**1 **^**(g)**	**Feed intake**^**2 **^**(g)**	**Feed efficiency (weight gain/feed intake)**
Control	1.40 ± 0.55	34.2	0.041 ± 0.016
FPH	2.60 ± 0.89*	38.6	0.067 ± 0.023

*Abbreviations*: *FPH* fish protein hydrolysate, *TNFα* tumor necrosis factor alpha.

^1^Mean values ± SD are shown (n = 5).

^2^Total feed intake in 14 days calculated per mouse is shown.

*indicates significantly different from control (Mann–Whitney, p < 0.05). Significance could not be calculated for the feed intake.

### Fatty acid composition in liver and WAT

**Figure 1:**
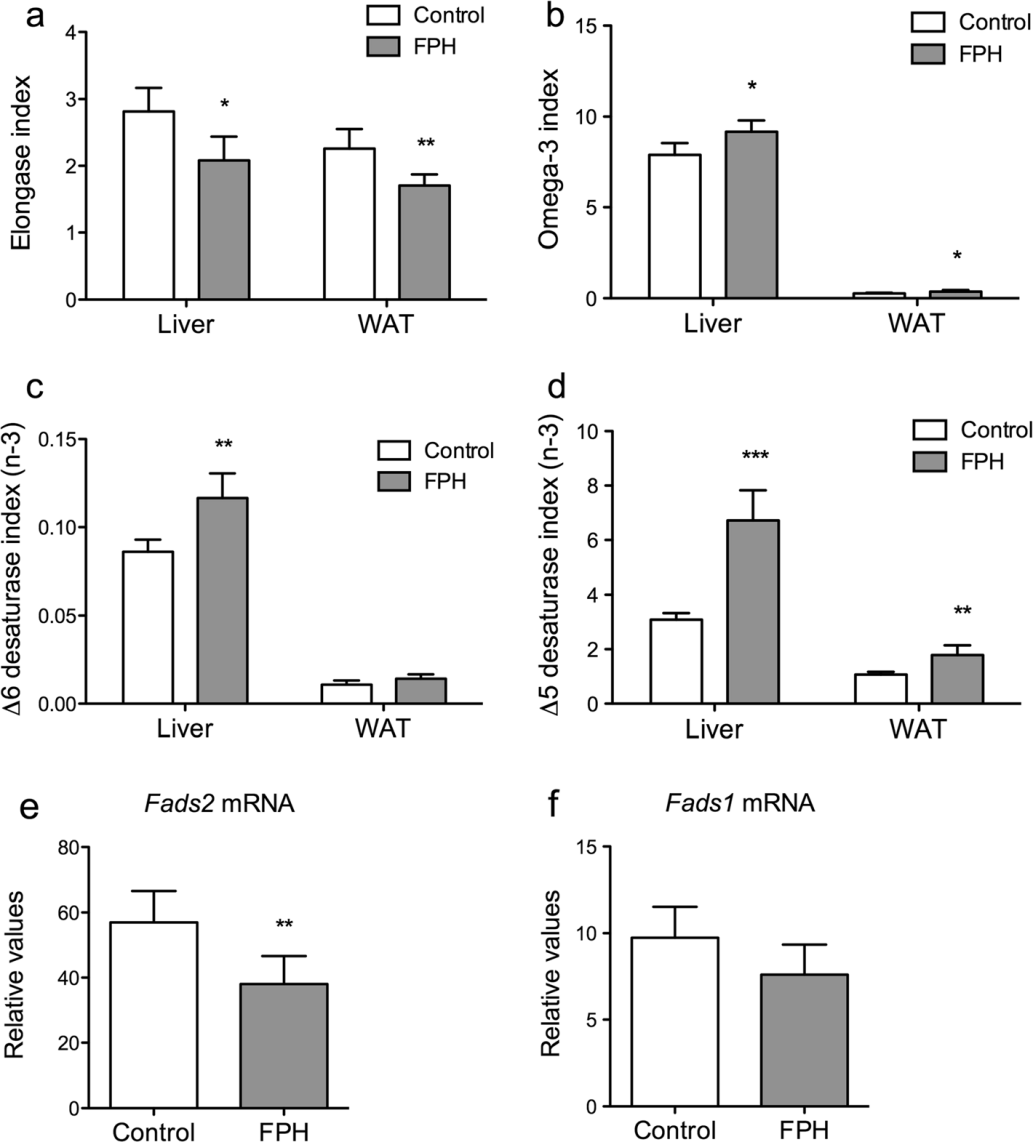
**The effect of fish protein hydrolysate on hepatic and white adipose tissue fatty acid composition.** hTNFα transgenic mice were given a 15% (w/w) fish protein hydrolysate (FPH) diet for two weeks, fasted over night, and fatty acid composition (wt%) was determined in liver and white adipose tissue (WAT), and gene expression was determined in liver. Elongase activity was calculated as an indirect index based on the n-3 PUFAs C20:3n-6/C18:3n-6 **(a)**. The omega-3 index was based on the sum of EPA (C20:5n-3) and DHA (C22:6n-3) **(b)**. An indirect index of ∆5 desaturase activity based on n-3 PUFAs was calculated as C20:5n-3/C20:4n-3 **(c)**. An indirect index of ∆6 desaturase activity based on n-3 PUFAs was calculated as C18:4n-3/C18:3n-3 **(d)**. Hepatic gene expression of D6 desaturase (*Fads2*) **(e)**. Hepatic gene expression of D5 desaturase (*Fads1*) **(f)**. Data are means ± SD (n = 5) of control (white bars) and FPH (grey bars). Values significantly different from control are indicated (*P < 0.05, **P < 0.01, ***P < 0.001).

The relative amounts of saturated fatty acids were higher in the ovarian WAT of TNFα-transgene mice fed FPH and this was due to an elevated level of C18:0 (Table 3). The total level of saturated fatty acids in the liver was unaffected, but a higher level of C16:0 was found after FPH feeding compared to control. The level of the monounsaturated fatty acid C18:1n-7 was lower both in liver and WAT in FPH-fed mice compared to control. This resulted in a lower elongase index (Figure 1a). Of the n-6 fatty acids, most were significantly lower in the liver of mice administered with FPH-diet, whereas hepatic C18:2n-6 was higher, giving no effect on total n-6 PUFA. The total hepatic level of n-3 fatty acid was greater in FPH-fed mice than in control TNFα mice. Significantly higher levels of EPA (C20:5n-3) and DPA (C22:5n-3) were found in both liver and WAT. In addition, the level of C18:3n-3 and C22:6n-3 was higher in the liver after FPH-feeding compared to casein-feeding. Altogether, the n-3 index was elevated in both liver and WAT after FPH administration (Figure 1b), as well as the ∆5 desaturase index for n-3 fatty acids and ∆6 desaturase index for n-3 fatty acids (Figure 1c and d). However, the hepatic ∆6 desaturase (*Fads2*) was significantly lower expressed at the gene level compared to control, while the ∆5 desaturase (*Fads1*) was insignificantly changed (Figure 1e and f).

**Table 3:** Fatty acid composition in liver and WAT of mice given control- or FPH-diets for 2 weeks and fasted over night

**Fatty acids % (w/w)**	**Liver**		**WAT**	
	**Control**	**FPH**	**Control**	**FPH**
∑ SFAs	34.57 ± 0.74	34.81 ± 0.87	32.35 ± 1.08	34.35 ± 1.15*
C16:0	21.42 ± 0.35	22.19 ± 0.49*	25.26 ± 0.83	26.13 ± 1.31
C18:0	12.02 ± 0.55	11.49 ± 0.57	4.61 ± 0.45	5.73 ± 0.55**
∑ MUFAs	25.43 ± 0.89	24.25 ± 1.31	48.62 ± 1.17	47.83 ± 2.35
C16:1n-7	1.03 ± 0.13	1.03 ± 0.12	4.74 ± 0.34	4.51 ± 0.46
C18:1*n-*7	1.60 ± 0.08	1.32 ± 0.09***	2.22 ± 0.08	2.04 ± 0.04**
C18:1*n-*9	21.77 ± 0.75	21.01 ± 1.15	40.35 ± 1.40	40.08 ± 5.53
∑ *n-*6 PUFAs	31.05 ± 0.59	30.52 ± 0.07	17.61 ± 1.38	16.21 ± 1.35
C18:2n-6	16.00 ± 0.54	17.03 ± 0.75*	16.70 ± 1.41	15.38 ± 1.30
C20:3*n-*6	0.90 ± 0.06	0.76 ± 0.05**	0.15 ± 0.01	0.12 ± 0.01*
C20:4*n-*6	12.96 ± 0.79	11.79 ± 0.57*	0.34 ± 0.04	0.30 ± 0.04
C22:4*n-*6	0.34 ± 0.01	0.25 ± 0.01***	0.07 ± 0.01	0.06 ± 0.01*
∑ *n-*3 PUFAs	8.73 ± 0.65	10.24 ± 0.68**	1.28 ± 0.10	1.49 ± 0.25
C18:3n-3	0.42 ± 0.03	0.50 ± 0.05*	0.89 ± 0.06	0.98 ± 0.14
C20:5*n-*3 (EPA)	0.29 ± 0.03	0.57 ± 0.10***	0.03 ± 0.01	0.06 ± 0.02**
C22:5*n-*3	0.29 ± 0.02	0.42 ± 0.02***	0.07 ± 0.01	0.09 ± 0.02*
C22:6*n-*3 (DHA)	7.60 ± 0.65	8.60 ± 0.60*	0.24 ± 0.03	0.32 ± 0.07
∑ *n-*3:∑ *n-*6 ratio	0.28 ± 0.35	0.34 ± 0.02**	0.07 ± 0.01	0.09 ± 0.01**

*Abbreviations*: *DHA* docosahexaenoic acid, *EPA* eicosapentaenoic acid, *FPH* fish protein hydrolysate, *MUFA* monounsaturated fatty acids, *PUFA* polyunsaturated fatty acids, *SFA* saturated fatty acids, *WAT* white adipose tissue.

Data are means ± SD (n = 5). Values significantly different from control are indicated (*P < 0.05,**P < 0.01,***P < 0.001).

### Anti-inflammatory index and plasma cytokines

**Figure 2:**
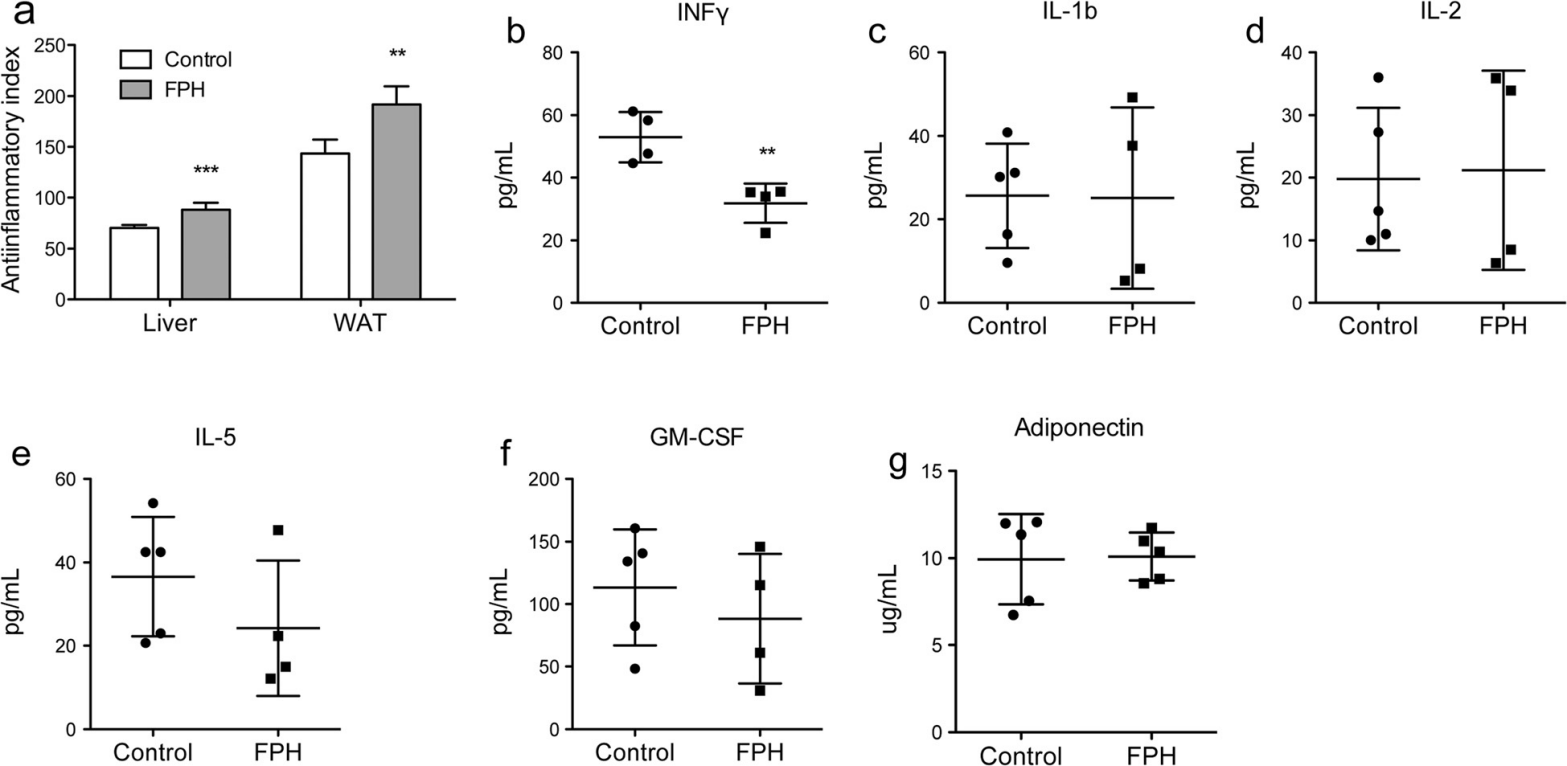
**A fish protein hydrolysate diet affected inflammation in TNFα mice.** hTNFα transgenic mice were given a 15% (w/w) fish protein hydrolysate (FPH) diet for two weeks and fasted over night. The anti-inflammatory index (n = 5) was calculated from wt% of fatty acids ((C22:5n-3 + C22:6n-3 + C20:3n-6 + C20:5n-3)/C20:4n-6)*100 **(a)**. The plasma level of INFγ **(b)**, IL-1b **(c)**, IL-2 **(d)**, IL-5 **(e)**, GM-CFS **(f)**, and adiponectin **(g)** was determined (n = 4–5) . Data are given as means ± SD, and values significantly different from control are indicated (**P < 0.01).

The fatty acid anti-inflammatory index was calculated from the levels of the anti-inflammatory fatty acids (C22:5n-3 + C22:6n-3 + C20:3n-6 + C20:5n-3) and the pre-inflammatory fatty acid arachidonic acid (C20:4n-6), and was found to be elevated both in liver and WAT after FPH-treatment (Figure 2a). In addition, the plasma level of interferon gamma (INFγ) was significantly decreased by the FPH diet compared to control (*P* = 0.0061) (Figure 2b). Interleukine-1 beta (IL-ib), IL-2, IL-5, and GM-CSF were not significantly different in FPH vs. control, nor were plasma adiponectin levels (Figure 2c-g).

### Hepatic fatty acid oxidation and synthesis

**Figure 3:**
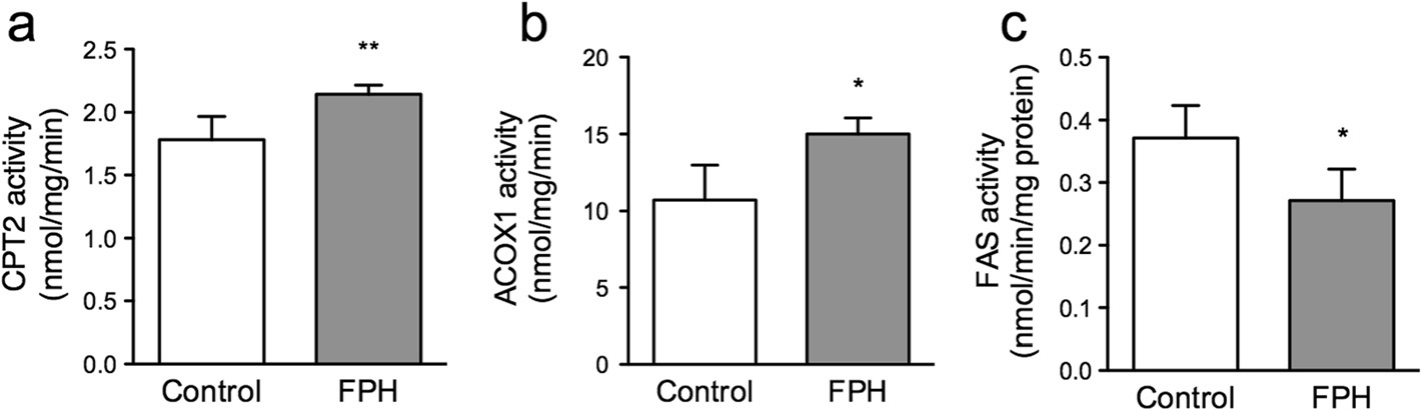
**The effect of fish protein hydrolysate on hepatic enzyme activity.** hTNFα transgenic mice were given a 15% (w/w) fish protein hydrolysate (FPH) diet for two weeks, and the hepatic enzyme activities of carnitine palmitoyl transferase-II (CPT-II) **(a)**, acyl-CoA oxidase 1 (ACOX1) **(b)**, and fatty acid synthase (FASN) **(c)** was measured. Data are given as means ± SD (n = 5), and values significantly different from control are indicated (*P < 0.05, **P < 0.01, ***P < 0.001).

The differences in fatty acid composition could be due not only to changes of the desaturases, but also enhanced mitochondrial and peroxiosmal fatty acid oxidation or synthesis in mice fed different diets. Both the hepatic activities of carnitine palmitoyltransferase II (CPT-II) and acyl-CoA-oxidase 1 (ACOX1), involved in mitochondrial and peroxisomal b-oxidation, respectively, were significantly higher in mice given the FPH diet (Figure 3a and b). CPT-I activity was similar in both groups (not shown). In addition, fatty acid synthesis, measured as fatty acid synthase (FAS) activity, was significantly lower in the FPH group compared to the control group (Figure 3c).

**Figure 4:**
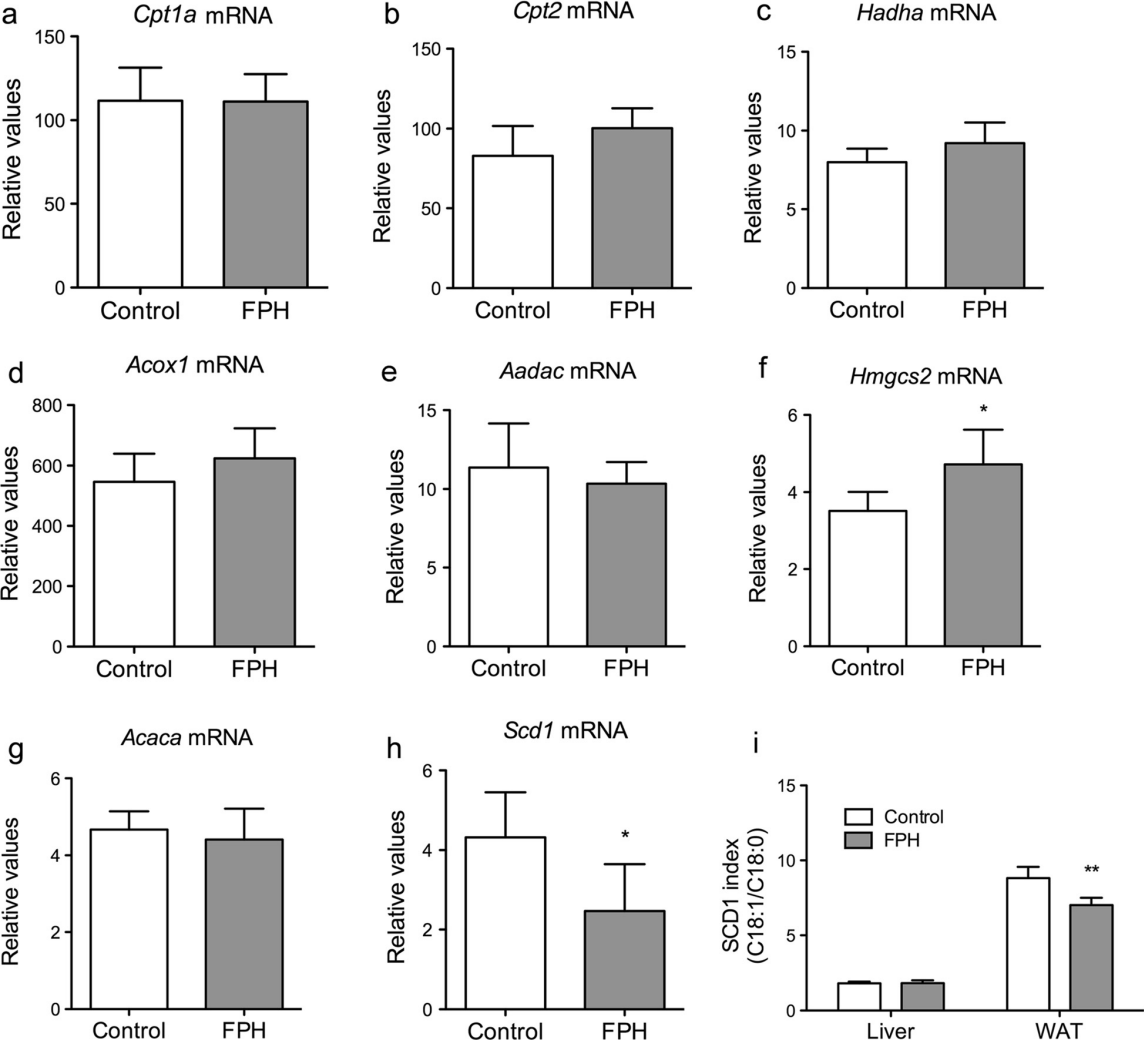
**The effect of fish protein hydrolysate on hepatic genes involved in fatty acid metabolism.** hTNFα transgenic mice were given a 15% (w/w) fish protein hydrolysate (FPH) diet for two weeks, fasted over night, and the hepatic expression of selected genes was analyzed. β-oxidation: carnitine palmitoyl transferase 1 (*Cpt1a*) **(a)**, carnitine palmitoyl transferase 2 (*Cpt2*) **(b)**, acyl-CoA oxidase 1 (*Acox1*) **(c)**, trifunctional enzyme (*Hadha*) **(d)**. Ketogenesis: HMG-CoA synthase 2 (*Hmgcs2*) **(e)**. Triacylglycerol lipase activity: arylacetamide deacetylase (*Aadac*) **(f)**. Lipogenesis: acyl-coA carboxylase (*Acaca*) **(g)**, (steroyl-CoA desaturase 1 (*Scd1*) **(h)**. An indirect index of hepatic and white adipose tissue (WAT) SCD1 activity was calculated as C18:1n-9/C18:0 **(i)**. Data are given as means ± SD (n = 5), and values significantly different from control are indicated (*P < 0.05, **P < 0.01, ***P < 0.001).

**Figure 5:**
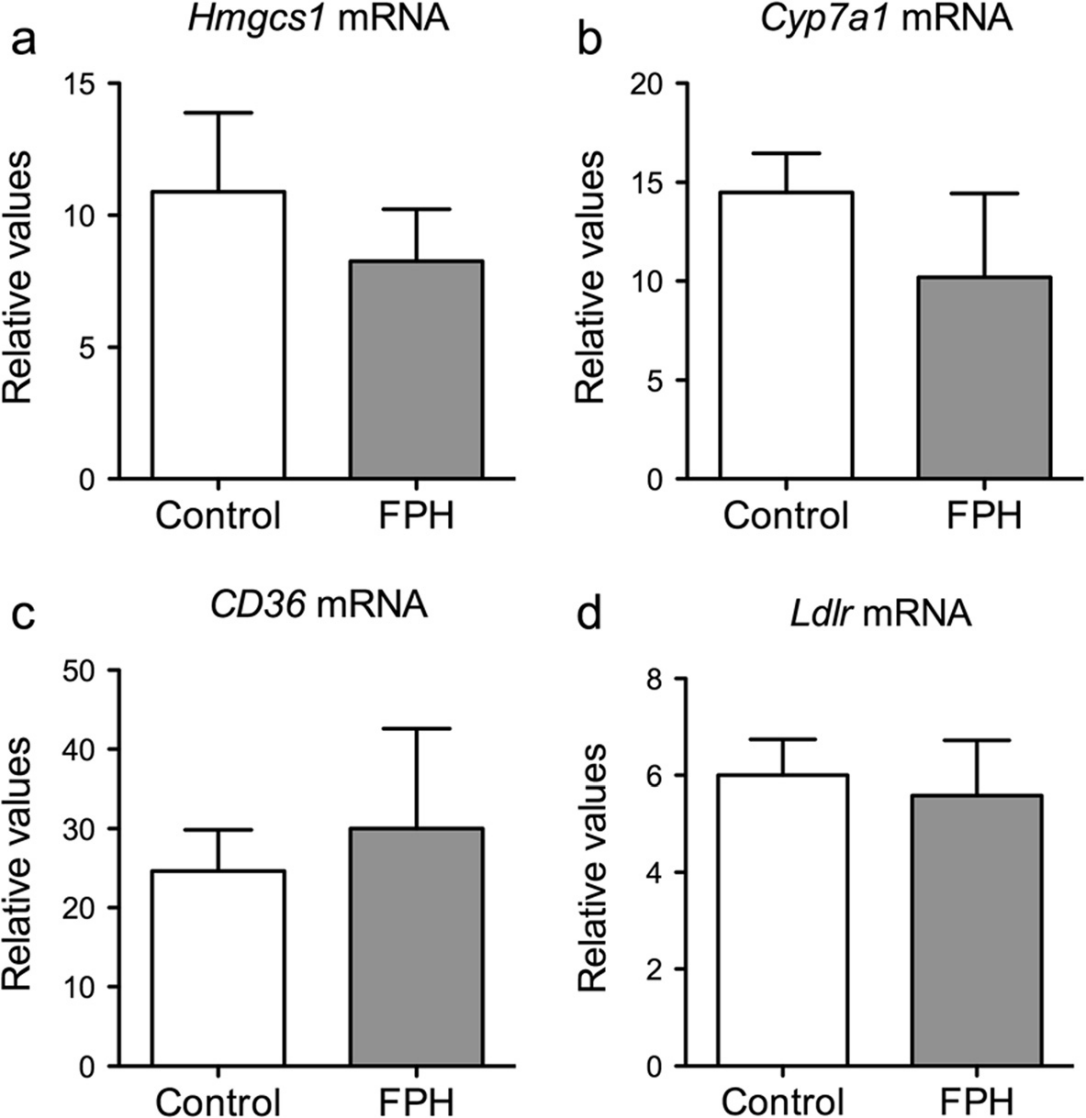
**No effect of fish protein hydrolysate on hepatic genes involved in cholesterol metabolism and lipid transport.** hTNFα transgenic mice were given a 15% (w/w) fish protein hydrolysate (FPH) diet for two weeks, fasted over night, and the hepatic expression of selected genes was analyzed: Cholesterol synthesis: HMG-CoA synthase 1 (*Hmgcs1*) **(a)**. Bile production: *Cyp7a1***(b)**. Fatty acid import: *CD36***(c)**. Lipoprotein import: low-density lipoprotein receptor (*Ldlr*) **(d)**. Data are given as means ± SD (n = 5), and values significantly different from control are indicated (*P < 0.05, **P < 0.01, ***P < 0.001).

On the mRNA level, no changes were observed in genes involved in β-oxidation (*Cpt1a*, *Cpt2*, *Acox1* or *Hadha*, Figure 4a-d), or in the release of fatty acids from TAG (arylacetamide deacetylase (*Aadac*), Figure 4e). However, HMG-CoA synthase 2 (*Hmgcs2*) gene expression was significantly higher in the FPH group compared to control (Figure 4f). The rate-limiting enzyme in fatty acid synthesis, acetyl-CoA carboxylase alpha (ACC), was not expressed differently at the mRNA-level (*Acaca*, Figure 4g), but the ∆9 desaturase (*Scd1*) was significantly lower in FPH treated mice (Figure 4h). The SCD1 index, based on the ratio between the SCD1-product and -substrate (C18:1n-9/C18:0), was significantly lower in FPH-fed mice only in WAT (Figure 4i), while C16:1n-7:C16:0 was unchanged (not shown). Neither selected genes involved in cholesterol synthesis and bile formation (*Hmgcs1* and *Cyp7a1*, respectively), nor genes involved in fatty acid- or low-density lipoprotein-import (*CD36* and low-density lipoprotein receptor (*Ldlr*), respectively) were affected by the FPH diet (Figure 5).

### Precursor of carnitine and carnitine esters in plasma

**Figure 6:**
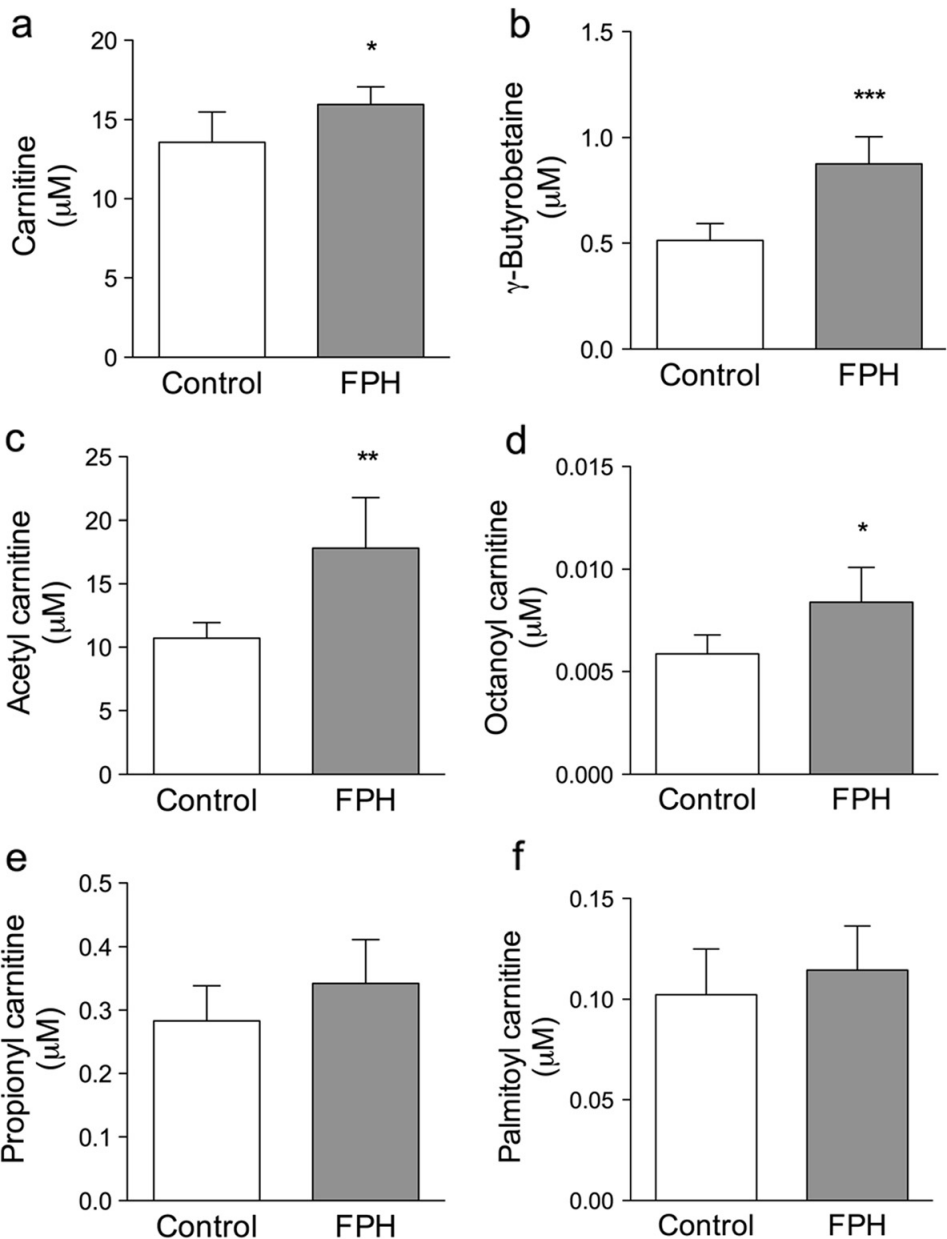
**A fish protein hydrolysate-diet increased plasma free carnitine, a carnitine precursor, and short-chained acylcarnitines.** Fasting plasma levels of free carnitine **(a)**, γ-butyrobetaine **(b)**, acetylcarnitine **(c)**, propionylcarnitine **(d)**, octanoylcarnitine **(e)**, and palmitoylcarnitine **(f)** after two weeks fish protein hydrolysate (FPH) treatment. Data are given as means ± SD (n = 5), and values significantly different from control are indicated (*P < 0.05, **P < 0.01, ***P < 0.001).

Carnitine is necessary to transport CoA-activated fatty acid into the mitochondria and out of the peroxisomes. It was of interest that both carnitine itself (Figure 6a) and the precursor of carnitine, γ-butyrobetaine (Figure 6b) were found at higher levels in plasma of FPH-fed mice compared to control-diet fed mice. Moreover, a higher level of acetylcarnitine (Figure 6c) was observed, and a small but significantly higher level of octanoylcarnitine (Figure 6d). Plasma levels of propionylcarnitine and palmitoylcarnitine were not significantly different between groups (Figure 6e and f).

## Discussion

Several investigations have reported an effect of inflammatory cytokines on lipid metabolism [23-26]. The target genes of PPARα, an important transcription factor regulating the fasting response, were downregulated in the liver of TNFα overexpressing mice [22]. As PPARα regulates both the lipid- and amino acid metabolism, we have in the present study investigated whether an FPH-diet can counteract TNFα-induced metabolic aberrations. We observed a significant effect on the hepatic and WAT fatty acid composition of FPH-fed mice, linked to effects on the gene expression or activity of genes involved in desaturation and lipogenesis. This demonstrates that casein- and salmon derived sources of protein differentially influence lipid metabolism in hTNFα mice.

The higher activity of CPT-II and ACOX1 accompanied with higher plasma levels of short-and medium carnitine esters in mice fed FPH suggest that this diet was able to improve the hepatic fatty acid oxidation capacities compared to a casein diet. Acetyl-CoA could potentially be used for keton body production, as the gene expression of the mitochondrial HMG-CoA synthase was upregulated. Alternatively, the observed higher level of plasma L-carnitine in FPH-fed mice could facilitate mitochondrial efflux of β-oxidation products. L-carnitine supplementation has previously been shown to relieve lipid overload and increase glucose sensitivity in obese mice [27].

A cholesterol lowering effect of FPH diets compared to casein diets have previously been observed in rodents, and may be due to decreased intestinal absorption concomitant with increased hepatic excretion of cholesterol and bile [28,29]. FPH has in some rodent studies also resulted in lower plasma triacylglycerol (TAG) levels [15,30]. It was therefore of importance to note that in this study FPH did not reduce plasma cholesterol or TAG levels. The reason for this is not clear, but one possibility is that the mitochondrial CPT-II activity was marginally higher with FPH mice than in control mice (20%) and that no significantly elevated gene expression of the PPARα activated genes *Cpt1a*, *Cpt2,* nor *Hadha* were observed. The observed lower lipogenesis with FPH vs. casein, similar to observations with vegetable protein diets in rats [13], was not sufficient to lower plasma TAG levels (Figure 3c). Also, no effect was seen on genes involved in cholesterol and bile production. It might be that a longer feeding period than two weeks is required to get a more pronounced effect on mitochondrial fatty acid oxidation and thereby overcome the hypertriglyceridemia in the TNFα mice. Indeed, in another experiment with male C57BL/6 normolipidemic mice treated for 6 weeks, the plasma TAG concentration was 28% lower in 15% FPH fed mice than in casein fed mice, while cholesterol was unchanged (RK Berge, data to be published). Interestingly, the male mice gained less weight with a higher feed intake than the control group, while in the current study the female FPH mice gained more weight than control mice. This might indicate sex-specific effects of FPH.

In the present study we show that the fatty acid composition was different in the liver and WAT of mice fed the FPH diet compared to the control diet, although the diets did not differ substantially in fatty acid composition. In the liver this can at least partly be due to higher mitochondrial and peroxisomal fatty acid oxidation. The ∆5 and ∆6 fatty acid indices were higher in mice fed FPH than casein, thus the changes in the fatty acid composition could also be due to differences in the activities of ∆5 and ∆6 desaturases. The hepatic gene expression of ∆6 desaturase was, however, lower than control whereas the mRNA level of ∆5 desaturase was identical after FPH administration. The lower gene expression levels could be a negative regulatory response to a higher level of unsaturated fatty acids. This is in agreement with a previous study demonstrating that in human WAT the gene expression of ∆5 and ∆6 desaturase was poorly reflected in the corresponding indices [31]. In contrast, the different SCD1 indices was linked to the gene expression level of *Scd1*[31]. Thus, the lower hepatic gene expression of *Scd1* in FPH-fed compared to casein-fed mice might explain the corresponding low SCD1-index in WAT from FPH-mice. Interestingly, SCD1 has been shown to regulate inflammation and stress in several cell types [25]. Also, a low SCD1 expression level has been indicated to protect against obesity and insulin resistance, while the opposite is true for high SCD1 levels [18,19]. Particularly, a high WAT 18:1/18:0 ratio has been linked to increased probability of insulin resistance in older men [31]. Thus, the observed reduction in the 18:1/18:0 ratio in WAT of FPH-fed mice could indicate effects on fatty acid denaturation beneficial to health.

As the FPH is a crude mix of hydrolysed peptides, further isolation of active peptides is necessary to identify the active component in this mix. There may also be an influence by dietary EPA and DHA in the FPH group. Analysis of the diets indicated only a small difference in the n-3/n-6 PUFA ratio between the diets. However, the EPA and DHA levels, although low, were increased in the FPH compared to the control diet due to the 2% remnant of fish oil in the protein hydrolysate (Table 4). Thus to identify the protein-specific effects, the EPA and DHA content should be kept constant in further studies.

**Table 4:** Amino acid composition, fatty acid composition, and vitamin D content of the experimental diets

**Diet groups**	**Control (wt%)**^**a**^	**FPH (wt%)**
Amino acids:		
ALA	3.01	6.97
ARG	3.14	5.23
ASP	7.27	8.51
GLU	22.14	16.42
GLY	1.85	8.21
HIS	2.60	2.32
HYP	0.00	1.54
ILE	4.88	3.74
LEU	9.17	7.51
LYS	7.92	8.22
MET	2.89	3.13
PHE	4.92	3.51
PRO	10.61	6.69
SER	5.37	4.56
TAU	0.00	2.10
THR	4.04	4.01
TYR	3.92	2.29
VAL	6.26	5.04
Fatty acids:		
AA	0.18	0.18
EPA	0.02	0.10
DPA	0.08	0.11
DHA	0.05	0.20
n-3 PUFA/ n-6 PUFA	0,10	0,12

^a^ Weight % (wt%) of total protein or fatty acids.

*Abbreviations*: *AA* arachidonic acid, *Ala* Alanine, *Arg* Arginine, *Asp* Aspartic acid, *DHA* docosahexaenoic acid, *DPA* docosapentaenoic acid, *EPA* eicosapentaenoic acid, *FPH* fish protein hydrolysate, *Glu* Glutamate, *Gly* Glycine, *His* Histidine, *Hyp* Hydroxyproline, *Ile* Isoleucine, *Leu* Leucine, *Lys* Lysine, *Met* Methionine, *Phe* Phenylalanine, *Pro* Proline, *Ser* Serine, *Tau* Taurine, *Thr* Threonine, *Tyr* Tyrosine, *Val* Valine.

## Conclusions

Treatment with FPH resulted in lower hepatic and WAT levels of long-chain n-6 fatty acids compared to a casein control. The anti-inflammatory fatty acid index was thereby increased both in liver and WAT. As the plasma level of INF-γ was reduced in the FPH-treated mice while other cytokines measured were unchanged, FPH may have an anti-inflammatory potential perhaps linked to its effect on fatty acid metabolism. The main effect of feeding TNFα mice a FPH diet was on fatty acid composition, lipogenesis, β-oxidation and acylcarnitines. This indicates that regulation of lipid metabolism is important for the bioactivity of protein hydrolysates of marine origin.

## Methods

### Transgenic mice

Female transgenic mice expressing human TNFα (hTNFα) were used (Taconic, Germantown, USA). This mouse line is generated in the strain C57BL/6, and express low levels of hTNFα [32]. The experiments were performed in accordance with, and under the approval of, the Norwegian State Board for Biological Experiments, the Guide for the Care and Use of Laboratory Animals, and the Guidelines of the Animal Welfare Act. The mice were divided into two experimental groups of five animals each with comparable mean body weight and were housed five animals per cage under constant temperature (22 ± 2°C) and humidity (55 ± 5%). They were exposed to a 12 h light–dark cycle (light from 07.00 to 19.00) and had unrestricted access to tap water and food. The mice were acclimatized to these conditions for one week before the start of the experiment.

Protein and fat of the feeding diets were from casein sodium salt from bovine milk, 20% w/w (Sigma-Aldrich Norway AS, Oslo, Norway), and lard, 21% w/w (Ten Kate Vetten BV, Musselkanaal, Netherlands), plus soy oil, 2% w/w (Dyets Inc., Bethlehem, PA, USA). In addition, in the intervention group part of the casein protein was substituted by fish protein hydrolysate obtained from Atlantic salmon (FPH, 15% w/w, supplied by Marine Bioproducts AS, Storebø, Norway) (Table 5). The n-3 PUFA/n-6 PUFA was similar in the two diets; however the EPA and DHA amount was 5 and 4 times higher, respectively, indicating an influence of the 2% fish oil in the FPH diet (Table 4). After two weeks of feeding, the mice were anaesthetized under over night fasting conditions by inhalation of 2% isoflurane (Schering-Plough, Kent, UK). Blood was collected by aortic puncture with 7.5% EDTA and immediately chilled on ice. Plasma was prepared and stored at −80°C prior to analysis. Ovarian WAT and liver were dissected, immediately N_2_-cryo freeze-clamped and stored at −80°C.

**Table 5:** Composition of the experimental diets

**Ingredients**	**Control (g/kg of diet)**	**FPH (g/kg of diet)**
Casein^a^	238.7	59.7
FPH^b^	-	165.2
Lard	230	226.7
Soy oil	20	20
Cornstarch	179	193
Dyetrose	132	132
Sucrose	100	100
Fiber	50	50
AIN-93G mineral mix	35	35
AIN-93 vitamin mix	10	10
L-Cysteine	3	3
Choline bitartrate	2.5	2.5
*tert*-Butylhydroquinone	0.014	0.014

*Abbreviations*: *FPH* fish protein hydrolysate.

The diets were isoenergetic and isonitrogenous and contained 20 g of protein per 100 g of diet.

^a^Casein consisted of 83.8% (w/w) protein and 0.2% fat.

^b^FPH consisted of 90.8% protein and 2% oil.

### Plasma and hepatic lipids

Liver lipids were extracted according to Blight and Dyer [33], evaporated under nitrogen and redissolved in isopropanol before analysis. Lipids were measured enzymatically in plasma samples and hepatic extracts on a Hitachi 917 system (Roche Diagnostics GmbH, Mannheim, Germany) using the triacylglycerol (GPO-PAP) and cholesterol kit (CHOD-PAP) from Roche Diagnostics, the free fatty acid (FFA) kit from DiaSys Diagnostic Systems GmbH (Holzheim, Germany), and the phospholipid kit from bioMerieux SA (Marcy l’Etoile, France).

### Hepatic and WAT fatty acid composition

Total hepatic and WAT fatty acid composition was analyzed as described previously [22]. The anti-inflammatory fatty acid index was calculated as ((C22:5n-3 + C22:6n-3 + C20:3n-6 + C20:5n-3)/C20:4n-6)*100 [34].

### Amino acid composition in the diet

The amino acids in the diets were determined after hydrolysis in 6 m-HCl at 110°C for 22 h and pre-derivatisation with phenylisothiocyanate according to the method of Cohen & Strydom [35]. The supernatant was filtered and amino acids were characterised by a Biochrom 20 plus amino acid analyser as previously described [29].

### Hepatic enzyme activities

The livers were homogenized and fractionated as described earlier [36]. The activities of carnitine palmitoyltransferase–II (CPT-II) [37], acyl-CoA oxidase 1 (ACOX1) and fatty acid synthase (FASN) were measured in the post-nuclear fraction as described by Skorve *et al.*[38].

### Plasma carnitine composition and cytokines

Free carnitine, short-, medium-, and long-chain acylcarnitines, and the precursors for carnitine, trimethyllysine and γ-butyrobetaine, were analysed in plasma using LC/MS/MS as described previously [39]. The cytokines INF-γ, IL-1b, IL-2, IL-4, IL-5, IL-10, IL-12, and GM-CST were assessed in a 96-well plate assay using custom made eight-plex kits (Millipore Corp., St. Charles, IL, USA). Cytokines IL-4, IL-10 and IL-12 did not give a result due to more than two samples below detection limit. Plasma adiponectin was measured using a single-plex kit (Millipore). The analysis was performed on undiluted plasma samples, in an overnight protocol according to the manufacturer’s recommendations, using the Bio-Plex 200 system (BioRad, Hercules, CA, USA).

### Gene expression analysis

Total cellular RNA was purified from frozen liver samples, and cDNA was produced as previously described [40]. Real-time PCR was performed with Sarstedt 384 well multiply-PCR Plates (Sarstedt Inc., Newton, NC, USA) on the following genes, using probes and primers from Applied Biosystems (Foster City, CA, USA): acetyl-CoA carboxylase alpha (*Acaca*, Mm01304277_m1), *Acox1* (Mm00443579), trifunctional protein, alpha subunit (*Hadha*, Mm00805228_m1), CD36 antigen (*CD36* (*Fat*), Mm00432403), *Cpt1a* (Mm00550438), *Cpt2* (Mm00487202), cytochrome P450, family 7, subfamily A, polypeptide 1 (*Cyp7a1*, Mm00484152), 3-hydroxy-3-methylglutaryl-Coenzyme A synthase 1 (*Hmgc1,* Mm00524111), *Hmgc2* (Mm00550050), low density lipoprotein receptor (*Ldlr,* Mm00440169), stearoyl-CoA desaturase 1 (*Scd1*, Mm00772290_m1), or Solaris qPCR Gene Expression Assays (Thermo Fisher Scientific Inc.,Waltham, MA, USA): fatty acid desaturase 1 (*Fads1*, AX-064722-00), *Fads2*/∆6 fatty acid desaturase (AX-049816-00), arylacetamide deacetylase (*Aadac*, AX-049058-00). Three different reference genes were included: 18 *s* (Kit-FAM-TAMRA (Reference RT-CKFT-18 s)) from Eurogentec, Belgium, glyceraldehyde-3-phosphate dehydrogenase (*Gapdh*, Mm99999915_g1) from Applied Biosystems, and ribosomal protein, large, P0 (*Rplp0*, Gene ID 11837) from Thermo Fisher Scientific. NormFinder was used to evaluate the reference genes, and data normalized to *18 s* are presented.

### Statistical analysis

Data sets were analyzed using Prism Software (Graph-Pad Software, San Diego, CA) to generate the figures and determine statistical significance. The results are shown as means with their standard deviations (SD). Student’s t-test and Mann–Whitney test, for parametric data and non-parametric data, respectively, were performed to evaluate statistical differences between the two groups. *P*-values < 0.05 were considered significant.

## Competing interests

The authors declare that they have no competing interest.

## Authors’ contributions

BB participated in the gene expression studies, analyzed and performed statistical analysis on all results and drafted the manuscript. CB participated in the design and coordination of the study. MSR participated in the enzyme activity analysis and gene expression studies. AS performed the carnitine and acylcarnitine analysis. PB performed the fatty acid analysis. JS participated in the design and coordination of the study. RKB conceived of the study, and participated in its design and coordination and helped to draft the manuscript. All authors read and approved the final manuscript.
